# Flexible thermochromic fabrics enabling dynamic colored display

**DOI:** 10.1007/s12200-022-00042-3

**Published:** 2022-09-29

**Authors:** Pan Li, Zhihui Sun, Rui Wang, Yuchen Gong, Yingting Zhou, Yuwei Wang, Xiaojuan Liu, Xianjun Zhou, Ju Ouyang, Mingzhi Chen, Chong Hou, Min Chen, Guangming Tao

**Affiliations:** 1 Wuhan National Laboratory for Optoelectronics and Sport and Health Initiative, Optical Valley Laboratory, Huazhong University of Science and Technology, Wuhan, 430074 China; 2 School of Computer Science and Technology, Huazhong University of Science and Technology, Wuhan, 430074 China; 3 School of Optics and Electronic Information, Huazhong University of Science and Technology, Wuhan, 430074 China; 4 State Key Laboratory of Material Processing and Die and Mould Technology, School of Materials Science and Engineering, Huazhong University of Science and Technology, Wuhan, 430074 China

**Keywords:** Thermochromic fibers, Fabric code, Information interaction, Wet spinning

## Abstract

Color-changeable fibers can provide diverse functions for intelligent wearable devices such as novel information displays and human–machine interfaces when woven into fabric. This work develops a low-cost, effective, and scalable strategy to produce thermochromic fibers by wet spinning. Through a combination of different thermochromic microcapsules, flexible fibers with abundant and reversible color changes are obtained. These color changes can be clearly observed by the naked eye. It is also found that the fibers exhibit excellent color-changing stability even after 8000 thermal cycles. Moreover, the thermochromic fibers can be fabricated on a large scale and easily woven or implanted into various fabrics with good mechanical performance. Driven by their good mechanical and physical characteristics, applications of thermochromic fibers in dynamic colored display are demonstrated. Dynamic quick response (QR) code display and recognition are successfully realized with thermochromic fabrics. This work well confirms the potential applications of thermochromic fibers in smart textiles, wearable devices, flexible displays, and human–machine interfaces.

**Graphical Abstract:**

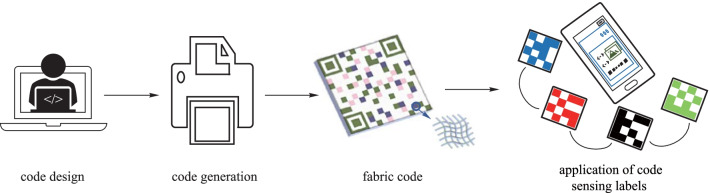

**Supplementary Information:**

The online version contains supplementary material available at 10.1007/s12200-022-00042-3.

## Introduction

Artificial intelligence and the Internet of Things is ushering in the age of information interaction. One supporting technology of intelligent information interaction is intelligent voice interaction. It is a human–machine interaction pattern based on machine vision [1], biometric identification and other auxiliary supporting technologies. These technologies include flexible display and sensing, both of which are in essence flexible interaction technology. Restricted by the existing technologies [2], traditional interaction interfaces tend to use regular hard planar screens. However, with flexible interaction [3], the display and sensing devices can operate normally with a certain range of deformation. Thus, flexible interaction no longer need a regular screen as the interaction interface, but the interaction object itself can be perceptible [4], displayable and dialogable [5]. Research in color-changeable fibers/fabrics has aroused much interest with the development of flexible interaction.

Most of the research on color-changeable fibers tends to introduce more intuitive color contents [6–8]. Therefore, if the color contents can respond to the external signal that carries information [9], they will not only make the fibers have a certain aesthetics, but also enable various potential applications of these fibers, such as drug release monitoring [10], temperature monitoring of human bodies [11], and display of time and temperature [12]. In addition, the color-changeable fibers can be woven into fabrics and make clothes with various colors [13], while maintaining the color and wearability of the cloth [14]. Due to the recent development in artificial intelligence, it is important to realize dynamic flexible interactive display by combining flexible color-changeable fiber technology [15] and intelligent interaction technology. Dynamic flexible interactive display may find applications in camouflage [16], wearable displays [17], vision sensors [18–20], temperature sensors [21], health detection, smart windows [22], tactile logic and many other fields [23].

This work aims to realize flexible colored display for wearable devices. We prepared stretchable, color-changeable thermochromic fibers with various colors based on the wet spinning process. It is demonstrated that, by encapsulating thermochromic microcapsules, the color of these fibers can be changed in the temperature range from − 15 °C to 70 °C. Moreover, the thermochromic fibers exhibit excellent color changeable stability even after 8000 thermal cycles. Large scale fabrication of thermochromic fibers are also realized and flexible thermochromic fabrics are obtained by warp and weft weaving or implanting into other fabrics. Driven by the unique characteristic of the developed fibers, dynamic colored display of thermochromic fabrics is realized and information interaction functionality is demonstrated. By fine-grained temperature control, thermochromic fabrics with dynamically changeable two-dimensional quick response (QR) code was successfully applied to information interaction. In the future, color-changeable fibers can be applied to the Internet of Things to create a more convenient smart environment.

## Experiment

### Preparation of thermochromic fibers

Thermoplastic polyurethanes (TPU, 9385A, Bayer Co. Ltd, China) particles (~ 1 mm) were washed in absolute ethanol and dried at 70 °C for about 4 h in a vacuum. Then the particles (40 g) were dissolved in dimethylacetamide (180 mL, DMAC, 95%, Sinopharm Chemical Reagent Co. Ltd, China) to form a solution. The polymer solution was mixed with thermochromic microcapsule (TCM, 10 g, ~ 9 μm, Chongyu Technology Co. LTD) (Additional file 1: Fig. S1) to form a thermochromic spinning stock solution. Thermochromic fibers were prepared by custom-made wet spinning machines (Changzhou, Lingxian Fabric Machinery Co., Ltd, China). The thermochromic spinning stock solution was injected into the coagulation bath (deionized water) at an advancing speed of 0.35 mL/min at indoor temperature. The thermochromic fibers passed through coagulation bath at a constant speed, which was controlled by the traction motor.

### Preparation of thermochromic display fabrics

The thermochromic display fabric with a pattern of “8” was prepared by warp and weft weaving using a small knitting machine (custom-made, China). Display fabrics containing other patterns (“HUST”, flower, and QR code) were prepared by using an embroidery machine (custom-made, China).

### Structure and performance characterization of thermochromic fibers and fabrics

The cross-section and surface of thermochromic fibers were characterized by scanning electron microscopy (EM-30 PLUS, COXEM). The tensile property measurement were conducted on a universal mechanical testing machine (CMT6103, MTS). The thermal stability of the thermochromic fabric was measured using a spectrophotometer (CR3, 3nh) (Additional file 1: Fig. S2). As shown in Additional file 1: Fig. S2, the whole testing setup was composed of a relay device, a spectrophotometer, and a heating device with temperature control. The chromatic aberration was calculated from the measured data in CIELAB color space. The reflection spectrum of thermochromic fabric was examined using an ultraviolet spectrometer (3600 plus, SHIMADZU).

### Design of APP for QR code recognition

In order to demonstrate the dynamic colored display functionality of thermochromic fabrics, an Android application (APP) for colored QR code recognition was developed. Communicating with a server, our APP can recognize a colored QR code and open the corresponding target APP in a smartphone. The detailed workflow is as follows. At first, our APP calls the camera of the smartphone to scan and record image information, and continuously transmits the collected image information to the server. Then, the server process and decode the image information for QR code recognition. The decoded QR code information will be compared with the data in an established database and the corresponding target APP will be found. The server then sends the information back to our APP and the target APP corresponding to the colored QR code will be opened.

## Results and discussion

### Structure characterization of thermochromic fibers

**Fig. 1:**
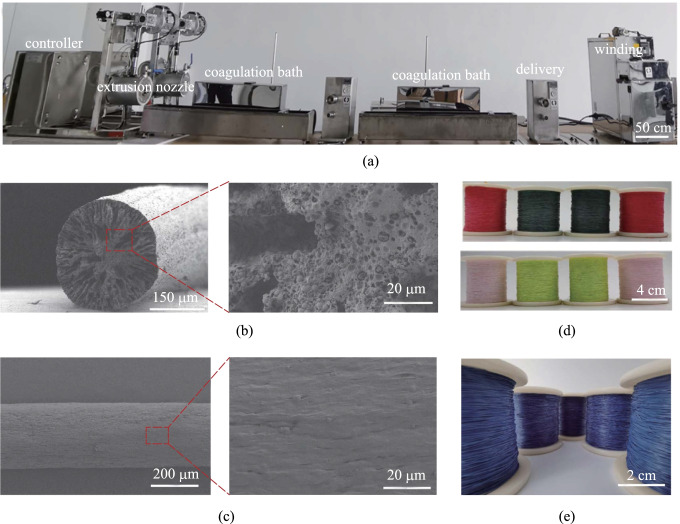
Fabrication and struction characterization of thermochromic fibers. **a** Continuous spinning device used to produce thermochromic fibers. **b** SEM images of cross section of thermochromic fibers. **c** SEM images of surface morphology of thermochromic microcapsules. **d** Photograph of thermochromic fibers with multi-color. **e** Large-scale production of thermochromic fibers

We fabricated the thermochromic fibers by a wet spinning process as shown schematically in Fig. 1a. Figure 1b, c displays a typical scanning electron microscopy (SEM) image of the cross section and surface morphology of thermochromic fibers, respectively. As can be seen from the enlarged SEM images, the thermochromic microcapsules were firmly embedded in the TPU, which ensured its excellent color change performance. In addition, the thermochromic fibers had a diameter of 0.3 mm, which could meet the requirements of wearable applications. The thermochromic fibers could be prepared with various colors such as red, green, pink, and yellow (Fig. 1d) and at large-scale (Fig. 1e).

### Performance of thermochromic fibers

**Fig. 2:**
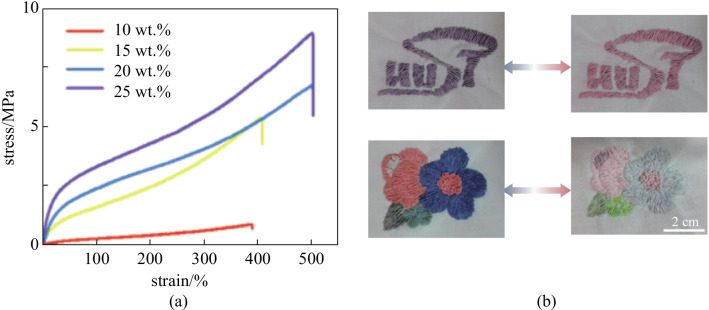
Performance of thermochromic fibers. **a** Stress/strain curves of thermochromic fibers at increasing TPU content, TPU percentage is increased from 10 wt.%, 15 wt.%, and 20 wt.% to 25 wt.%. **b** Display fabrics with different patterns (“HUST” and a flower) and their colors at 31 °C and 55 °C (scale bar: 2 cm)

Mechanical properties are crucial for the practical application of thermochromic fibers in the textile field. As shown in Fig. 2a, the maximal strain was affected by the TPU content. The less TPU, the lower mechanical strength and the smaller maximum strain. When the TPU percentage was decreased from 25 wt.% to 20 wt.%, 15 wt.% and 10 wt.%, the maximal strain was decreased from 503 to 502%, 410% and 391%, and the tensile strength at break was decreased from 8.9 MPa to 6.7, 5.3, and 0.8 MPa, respectively. Balancing between the ease of preparation and the performance of the fiber, a color-changeable fiber with TPU percentage of 20 wt.% was selected. The fiber could be stretched to 500% of the original length (Additional file 1: Fig. S3).

The weaveability and color-changing capability of thermochromic fibers are very important for their practical applications. We demonstrated that the as-prepared thermochromic fibers could be woven into stretchable fabrics. A weaving machine was used to weave the fibers with commercial fiber materials, such as wool, to produce clothing with designed patterns, as shown in Fig. 2b. We found that, the color of the patterns could be easily adjusted by changing the temperature of the environment (Additional file 2: Movie S1, Additional file 3: Movie S2).

### Performance of thermochromic fabrics and fabric dynamic displays

**Fig. 3:**
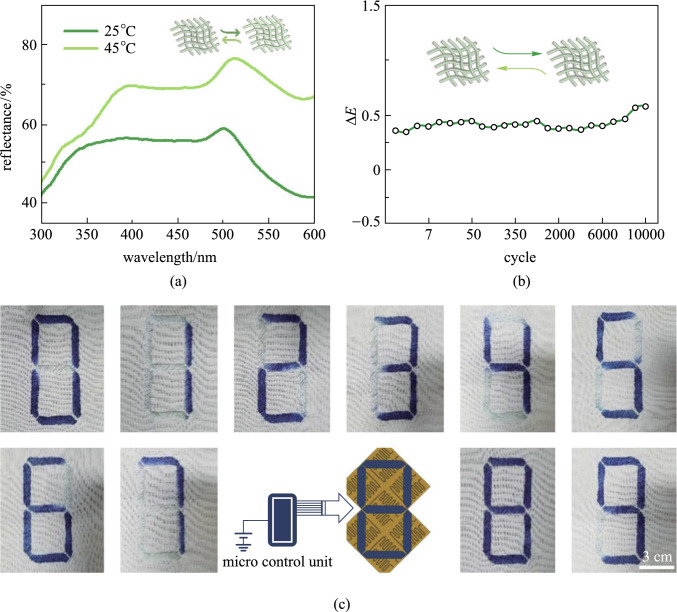
Performance of thermochromic fabrics and fabric dynamic displays. **a** Reflection spectra of the thermochromic fabric at the temperature of 25 °C (dark green curve) and 45 °C (light green curve), respectively. **b** Relative colorimetric response changes over 10,000 thermal cycles between 25 °C and 45 °C. **c** Thermochromic fabric displaying numbers from 0 to 9, with color change temperature of 55 °C (scale bar: 3 cm)

Reflectance spectrum of thermochromic fabric is critical for measuring color changes in the fabric. As shown in Fig. 3a, when the color of thermochromic fabric changed from dark green to light green, the peak reflectance increased noticeably. It is found that the reflectance at 510 nm increased from 58.12% to 77.18%. A test of another thermochromic fabric with the color changing from dark blue to light blue was also conducted (Additional file 1: Fig. S4). The color changes at different temperatures were evaluated using the definition of color difference suggested by National Bureau of Standards of USA [24]. The total color difference between the samples before and after discoloration was denoted as Δ*E* (Additional file 1: Table S1). To quantitatively evaluate the reversible color change properties and the stability of thermochromic fabrics, the color changes of the thermochromic fabrics with cyclic change of environmental temperature between 25 °C and 45 °C are recorded and the results are shown in Fig. 3b. It can be seen that $\Delta E$ fluctuates within a narrow range and the most value of $\Delta E$ was less than 0.5 over 8000 thermal cycles, this indicated that the physicochemical properties of the thermochromic microcapsules were stable within the working temperature range [24, 25]. With Temperature control, it is demonstrated that the patterns on the thermochromic fabric can be changed dynamically. As an example, we made a fabric that can display numbers from 0 to 9 (Additional file 4: Movie S3). To fulfill this task, thermochromic fibers and the commercial wool were woven into fabric with a pattern of “8”, which consisted of seven independent parts and the temperature of each part could be separately adjusted to 55 °C (Fig. 3c).

### Colored QR code on fabric for information interaction

**Fig. 4:**
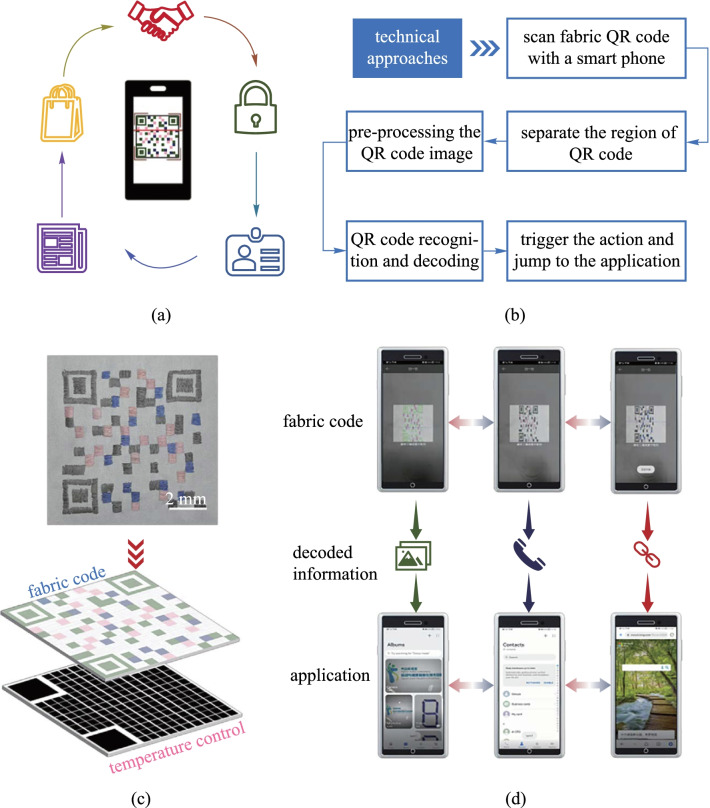
QR code on fabric for information interaction. **a** Colored fabric QR codes for various applications. **b** Basic flow scheme of colored QR code recognition. **c** Schematic of a heating scheme for dynamic fabric display. **d** Different information acquired from one fabric displaying three colored patterns at different temperatures

A fabric with colored pattern can be used to transfer information, which enables an enormous range of applications. The information could involve text, website links, images, and videos. Thus, we design a fabric displaying colored QR code based on the thermochromic fibers. A colored QR code is different from ordinary black-and-white QR code in that different patterns can be obtained by temperature controlling (Additional file 5: Movie S4). The fabric QR code can find applications in many circumstances, such as social contact, information security, identification, information transfer and electronic payment (Fig. 4a). The recogniziton of the colored fabric pattern is based on a specially designed color recognition algorithm. Figure 4b shows the basic flow scheme. To fulfill dynamic display, the whole pattern was divided into several zones, where the temperature can be controlled independently. Figure 4c shows the schematic diagram of the heating method. Three different QR codes can be obtained by zoned temperature control, which corresponds to different APPs, respectively (Additional file 6: Movie S5). The scan results were shown in Fig. 4d.

## Conclusion

In summary, color reversible and stretchable thermochromic fibers with thermochromic microcapsules are fabricated based on the wet spinning process. The so-obtained thermochromic fibers exhibit good mechanical properties and excellent color change stability even after 8000 thermal cycles between 25 °C and 45 °C. They can be fabricated on a large scale and easily woven or implanted into various fabrics. The fabrication strategy of thermochromic fibers can be also applied to fabrication of other stretchable functional composite fibers. More importantly, we fabricate fabrics with color-changeable patterns, which could provide functions of both static and dynamic image display for information interaction. Colored QR code recognition was also demonstrated using a specially designed APP. We expect that, with universal accessibility of the colored QR code fabrics, more possibilities can be opened up in the Internet of Things, especially regarding to intelligent information interaction and information security protection.

## Supplementary Information

**Additional file 1: Table S1** Relationship between the color-change value $\Delta E$ and traditional chromatic aberration (CA) observed by the naked eye**. Fig. S1** SEM images of thermochromic microcapsules. The average particle size of thermochromic microcapsules is from 3 to 10 μm. **Fig. S2 **(a) Device to test the color-changeing stability of thermochromic fabrics. (b) Initial color of thermochromic fabric before heating. (c) Color of the thermochromic fabric when heated for a set period of time (scale bar 3 cm). **Fig. S3** Tensile property test of the thermochromic fiber. The stretch ratios are 100% (a), 200% (b), 300% (c), 400% (d), and 500% (e) respectively. **Fig. S4** Reflectance spectrum of thermochromic fabric. Corresponding to the change of the spectral peak, the surface color of the thermochromic fibers can be switched from dark bule to light blue when the transition temperature of 55 °C (light blue curve) is reached. The peak intensity decreases noticeably, and the reflectance at 470 nm decreases from 18.89% to 8.13%.

**Additional file 2: Movie. S1** The display of a “HUST” pattern on a fabric based on thermochromic fibers (The temperature was changed from 25 to 31 °C).

**Additional file 3: Movie. S2** The display of a “Flower” pattern on a fabric based on thermochromic fibers (The temperature was changed from 25 to 55 °C).

**Additional file 4: Movie. S3** The dynamic display of a color changeable fabric with a pattern of “8” (The temperature was changed from 25 to 55 °C).

**Additional file 5: Movie. S4** The display of a QR code pattern on a fabric based on thermochromic fibers (The temperature was changed from 25 to 65 °C).

**Additional file 6: Movie. S5** Three different information obtained with the color-changing QR code.
