# Supplementary material for: Flexible thermochromic fabrics enabling dynamic colored display
Source: Front Optoelectron. 2022 Sep 29;15(1):40. doi: 10.1007/s12200-022-00042-3 (PMC9756210; doi:10.1007/s12200-022-00042-3)
Supplement: Supplementary file 1 — Additional file 1: Table S1 Relationship between the color-change value $\Delta E$ΔE and traditional chromatic aberration (CA) observed by the naked eye. Fig. S1 SEM images of thermochromic microcapsules. The average particle size of thermochromic microcapsules is from 3 to 10 μm. Fig. S2 (a) Device to test the color-changeing stability of thermochromic fabrics. (b) Initial color of thermochromic fabric before heating. (c) Color of the thermochromic fabric when heated for a set period of time (scale bar 3 cm). Fig. S3 Tensile property test of the thermochromic fiber. The stretch ratios are 100% (a), 200% (b), 300% (c), 400% (d), and 500% (e) respectively. Fig. S4 Reflectance spectrum of thermochromic fabric. Corresponding to the change of the spectral peak, the surface color of the thermochromic fibers can be switched from dark bule to light blue when the transition temperature of 55 °C (light blue curve) is reached. The peak intensity decreases noticeably, and the reflectance at 470 nm decreases from 18.89% to 8.13%. [file 12200_2022_42_MOESM1_ESM.docx]

**Supporting Information**

**Flexible Thermochromic Fabrics enable Dynamic Colored Display**

Pan LI^†^, Zhihui SUN^†^, Rui WANG^†^, Yuchen GONG, Yingting ZHOU, Yuwei WANG, Xiaojuan LIU, Xianjun ZHOU, Ju OUYANG, Mingzhi CHEN, Chong HOU, Min CHEN, Guangming TAO(🖂)

E-mail: [tao@hust.edu.cn](mailto:tao@hust.edu.cn)

**Table S1** Relationship between the color-change value $\Delta E$ and traditional chromatic aberration (CA) observed by the naked eye

| Range of $\Delta E$ | Chromatic aberration observed by the naked eye |
| --- | --- |
| 0—0.25 | Very small or no CA, the two colors are ideally matched |
| 0.25—0.5 | Tiny CA, which is acceptable in most applications |
| 0.5—1.0 | Small to medium CA, which is acceptable in some applications |
| 1.0—2.0 | Medium CA, which is acceptable in specific applications |
| 2.0—4.0 | Obvious CA, which is acceptable in specific applications |
| Over 4.0 | Very large CA, which is unacceptable in most applications |

**Table S1** represents the degree of color change when $\Delta E$ is in different range. The value can be calculated by the following formula：

$\Delta E=\sqrt{\left( \Delta L \right)^{2}+\left( \Delta a \right)^{2}+\left( \Delta b \right)^{2}}$.

The higher the value of$L$, the higher the brightness of the color. Increasing L represents the color changes from the dark to bright. The change of $a$ from a negative value to a positive value represents a change in color from green to red. The change of$b$ from a negative value to a positive value represents a change in color from blue to yellow.


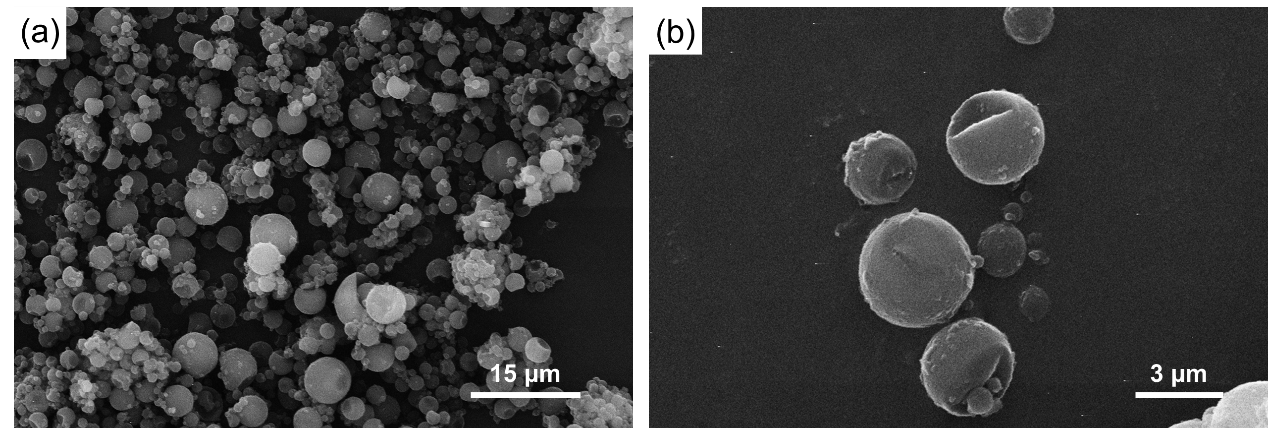


**Fig. S1** SEM images of thermochromic microcapsules. The average particle size of thermochromic microcapsules is from 3 to 10 μm.


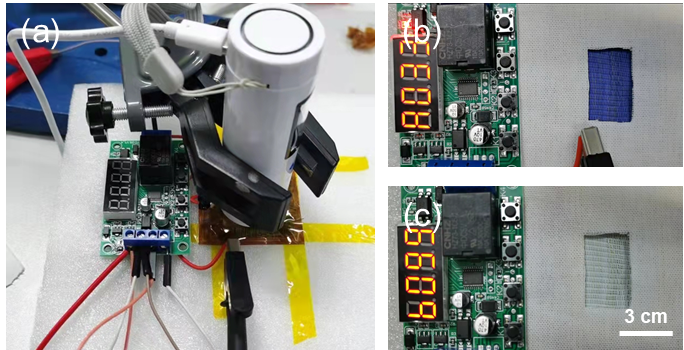


**Fig. S2** (a) Device to test the color-changeing stability of thermochromic fabrics. (b) Initial color of thermochromic fabric before heating. (c) Color of the thermochromic fabric when heated for a set period of time (scale bar 3 cm).


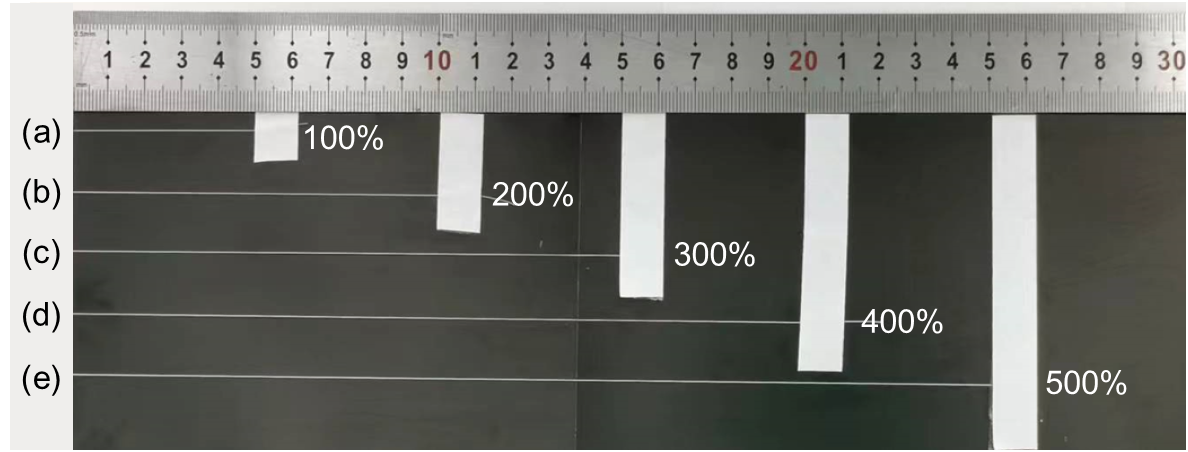


**Fig. S3** Tensile property test of the thermochromic fiber. The stretch ratios are 100% (a), 200% (b), 300% (c), 400% (d), and 500% (e) respectively.


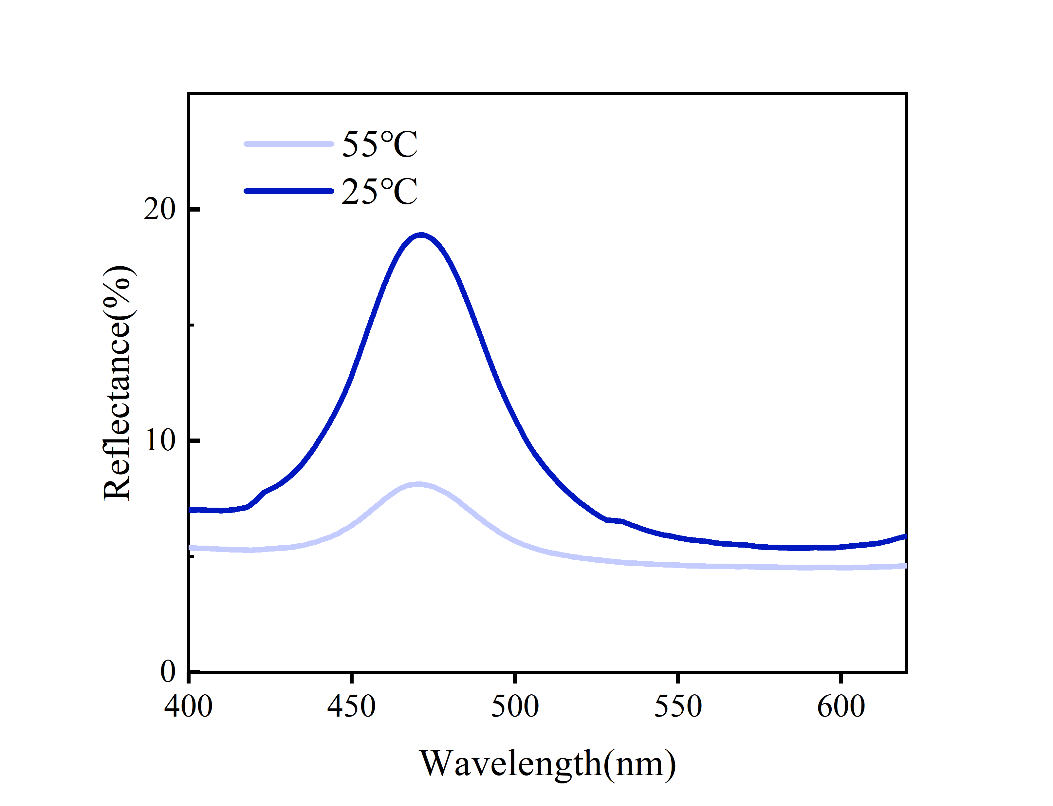


**Fig. S4** Reflectance spectrum of thermochromic fabric. Corresponding to the change of the spectral peak, the surface color of the thermochromic fibers can be switched from dark bule to light blue when the transition temperature of 55 °C (light blue curve) is reached. The peak intensity decreases noticeably, and the reflectance at 470 nm decreases from 18.89% to 8.13%.

**Caption for Supplementary Movie**

**Movie. S1** The display of a “HUST” pattern on a fabric based on thermochromic fibers (The temperature was changed from 25 to 31 °C).

**Movie. S2** The display of a “Flower” pattern on a fabric based on thermochromic fibers (The temperature was changed from 25 to 55 °C).

**Movie. S3** The dynamic display of a color changeable fabric with a pattern of “8” (The temperature was changed from 25 to 55 °C).

**Movie. S4** The display of a QR code pattern on a fabric based on thermochromic fibers (The temperature was changed from 25 to 65 °C).

**Movie. S5** Three different information obtained with the color-changing QR code.
